# Real-Time Monitoring and Fault Diagnosis of a Low Power Hub Motor Using Feedforward Neural Network

**DOI:** 10.1155/2016/7129376

**Published:** 2015-12-27

**Authors:** Mehmet Şimşir, Raif Bayır, Yılmaz Uyaroğlu

**Affiliations:** ^1^Sakarya University Institute of Natural Sciences, 54187 Sakarya, Turkey; ^2^Karabük University Technology Faculty, 78050 Karabük, Turkey; ^3^Sakarya University Engineering Faculty, 54187 Sakarya, Turkey

## Abstract

Low power hub motors are widely used in electromechanical systems such as electrical bicycles and solar vehicles due to their robustness and compact structure. Such systems driven by hub motors (in wheel motors) encounter previously defined and undefined faults under operation. It may inevitably lead to the interruption of the electromechanical system operation; hence, economic losses take place at certain times. Therefore, in order to maintain system operation sustainability, the motor should be precisely monitored and the faults are diagnosed considering various significant motor parameters. In this study, the artificial feedforward backpropagation neural network approach is proposed to real-time monitor and diagnose the faults of the hub motor by measuring seven main system parameters. So as to construct a necessary model, we trained the model, using a data set consisting of 4160 samples where each has 7 parameters, by the MATLAB environment until the best model is obtained. The results are encouraging and meaningful for the specific motor and the developed model may be applicable to other types of hub motors. The prosperous model of the whole system was embedded into Arduino Due microcontroller card and the mobile real-time monitoring and fault diagnosis system prototype for hub motor was designed and manufactured.

## 1. Introduction

The increasing needs of industry and daily life make electrical energy and ergo electrical motors more and more important. Electrical motors are the electrical machines which convert electrical energy into mechanical energy. There have been many studies about design, control, and overcoming the problems of electrical motors to improve them [1–5]. In spit of successful studies and improvements about electrical motors, owing to the thermal, electrical, and mechanical attritions, ageing and faults are unavoidable. Faults can result in serious performance degradation and eventual system failures if they are not properly detected and handled [6]. Early detection of abnormalities in the motor will help to avoid expensive failures. Operators of electric drive systems are under continuous pressure to reduce maintenance costs and prevent unscheduled downtime that result in lost production and financial losses [7]. Condition-based maintenance strategies reduce faults and increase the time between planned shutdowns for planned maintenance. They minimize failures and reduce maintenance durations and operational costs. Nevertheless, sometimes even failures are unavoidable; reducing them to a limited ratio is a big advantage. This situation makes condition monitoring and fault diagnosis more important.

The issues of preventive and condition-based maintenance, online monitoring, system fault detection, diagnosis, and prognosis are of increasing importance [8]. Various studies were presented about condition monitoring, fault diagnosis, and detection using intelligent systems and mostly neural networks over the years. Talebi et al. suggested a fault detection system using dynamic recurrent neural networks by utilizing a comprehensive dynamic model which contains both mechanical and electrical components of the wind energy conversion systems [9]. A winding-function-based method was developed by Joksimovic and Penman, for modelling polyphase cage induction motors with interturn short circuit in machine stator winding [10]. Uçar et al. detected alternator failures using fuzzy logic and an artificial neural network [11]. A fault diagnosis system was presented by Bayır and Bay for a serial wound starter motor based on a multilayer feedforward artificial neural network [12]. Bayır and Bay presented a fault diagnosis and monitoring system of serial wound pre-engaged starter motors by using a Kohonen neural network [13]. Fault monitoring and diagnosis algorithms were proposed by Huang and Yu using an observer model for induction motors. The monitoring technique is applied to send out a warning signal when a fault is detected [14]. Youn et al. described the initial step taken to develop an online diagnosis method for identifying broken rotor bar faults using flux signals [15]. Akın et al. reported that the reference frame theory approach could successfully be applied to real-time fault diagnosis of electric machinery systems as a powerful toolbox to find the magnitude and phase quantities of fault signatures with good precision as well [16]. A method to detect and diagnose induction motor broken bars faults was presented by Bouzid et al. in an early stage based on monitoring suitable features by a feedforward multilayer perceptron neural network [17]. A brief review of bearing, stator, rotor, and eccentricity-related faults and their diagnosis techniques was prepared by Nandi et al. [18]. Singh and Kazzaz used C++ and MATLAB programs for the development of the feedforward neural network with backpropagation algorithm for induction motor diagnostic and monitoring [19]. Model-based fault detection methods were analysed by Isermann, like parameter estimation, observers and parity equations, and fault-diagnosis methods, like classification and inference methods, and presented examples about model-based fault detection [20]. Nan et al. proposed a knowledge-based fault diagnosis method, which uses the valuable knowledge from experts and operators, as well as real-time data from a variety of sensors. Fuzzy logic is also used to make inferences based on the acquired information (real-time data) and knowledge [21]. Uysal and Bayır detected faults in switched reluctance motors and diagnosed them in real-time with the Kohonen neural network [22]. A fuzzy logic based fault detection system was developed by Bayır and Bay for implementation on emergency vehicles [23]. Bayır developed a graphical user interface software for real-time condition monitoring and fault diagnosis of serial wound starter motors using a Learning Vector quantization neural network [24]. Vijay et al. evaluated seven wavelet based denoising schemes based on the performance of the artificial neural network (ANN) and the support vector machine (SVM), for the bearing condition classification [25]. A neurofuzzy-based perspective to the automation of diagnosis and location of stator-winding interturn short circuits was presented by Awadallah et al. in CSI (current source inverter) fed brushless DC motors [26]. Matić et al. detected the broken rotor bar faults for induction motors with a feedforward backpropagation neural network [27]. An artificial neural network based fault diagnosis of permanent magnet synchronous motors was presented by Moosavi et al. for classifying interturn short circuit faults in stator winding by using a multilayer feedforward neural network [28]. Budiharto presented a new method of vision-based surveillance robot with obstacle avoidance capabilities for general purpose robots in indoor environments. Algorithms of neural network for obstacle avoidance were implemented in the robot [29].

Although the usage of hub motors is being spread day by day and the number of studies presented regarding hub motors is increasing, there were few studies undertaken about faults of system with hub motors. Rajagopal and Sathaiah optimized a 30 W, 48 V, and 310 RPM (revolutions per minute), PM (permanent magnet), and BLDC (brushless direct current) hub motor using the developed CAD (computer aided design) program. The design variables, such as flux density in the air gap and iron, slot space factor, stack length of the motor, air gap length, and number of magnet poles, are assumed. Output of the developed CAD program gave the design data and the same is validated by FE analysis [30]. Gruber et al. optimized a hub motor for an electric scooter. The power and torque density of the hub motor were improved by a multidimensional generic optimization which was achieved using a 2D finite element program [31]. Chanpeng and Hachanont designed and improved an efficient hub motor by means of basic mathematical equations of a hub motor and experimental studies [32]. The integrated Giant Magneto Resistance based steering wheel angular sensor was developed by Hao et al. for vehicles with hub motors [33]. Dadashnialehi et al. proposed a sensorless antilock braking system (ABS) that eliminates the need for the installation of separate conventional ABS sensors and saves the costs associated with the installation and maintenance of those sensors for electrical vehicles with hub motors [34]. An in-wheel electric motor prototype was designed by Çakır and Sabanovic for experimental purposes [35]. A fault diagnosis system was presented by Tashakori and Ektesabi to detect switch faults of three phases VSI (Voltage Source Inverter) drive of BLDC motor with a closed-loop control scheme [36]. Ifedi et al. presented studies of a fault-tolerant concept for the design of hub motors and focused on achieving a high torque density and the ability to sustain an adequate level of performance following a failure. They simulated a series of failures and then compared them with experimental tests on a demonstrator motor [37].

The main specification of this study is to achieve a comprehensive fault diagnosis of all of the possible faults of a hub motor by using just one fault diagnosis system design in a style which has never been approached before. This is not a model-based system and is not based on model simulation results. It is based on an actual, operating hub motor, by real-time measured data and real-time fault diagnosis on a real motor. This study presents the designing of the artificial feedforward backpropagation neural network based real-time monitoring and fault diagnosis system and manufacturing of an applicable prototype of the system which was embedded into the microcontroller circuit for mobile applications. For this purpose, a test set for a hub motor was designed. Seven main parameters of hub motor were received and stored for normal conditions and all possible faults to compose the data set. The most suitable neural network was chosen by research and optimized with various neuron numbers through several tests. 80% of the data set was used for training and 20% was used for testing as separated homogenously. The artificial feedforward backpropagation neural network was designed and trained with training data, then tested with training data, and then also tested with testing data which was not used during the training process. Success percentage tests were applied offline at first by a coded MATLAB function embedded model which was designed for the test process and applied data set sequentially to the designed system. After this the success percentage tests were applied once more online and in real-time with a real operating hub motor. The tested and successful whole model was embedded into a microcontroller card. Finally, mobile and applicable prototype of the real-time monitoring and fault diagnosis system of a hub motor was designed and manufactured.

The structure of this paper is organised as follows.  Section 2 introduces the hub motor.  Section 3 presents the designed test set and data acquisition approach.  Section 4 explains the construction and training of the artificial neural network.  Section 5 expresses real-time fault diagnosis. Results and conclusions are given in Section 6.

## 2. Hub Motor

**Figure 1:**
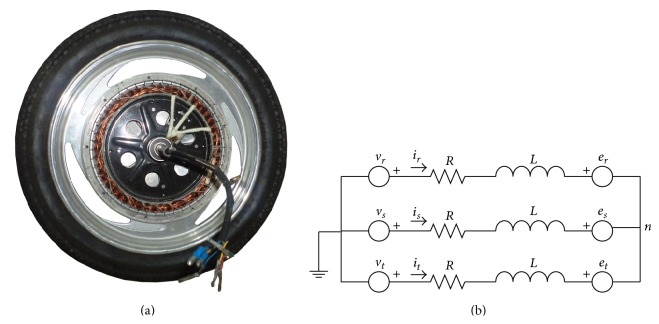
(a) Hub motor and (b) electrical equivalent circuit of hub motor.

Real-time monitoring and fault detection or diagnosis of the systems provides respectable advances for users of the concerned system or device. In this study, a three-phase, Y-connected, 48 V DC hub motor was used with a nominal power of 250 W. The motor has a maximum speed of 350 RPM and was energized with a 48 V DC power supply. The brushless direct current hub motor is the most suitable choice for electrical vehicles due to its physical structure, torque characteristics, robustness, and low mechanical losses. Hub motors are PM BLDC motors with inversely located rotor and stator placements according to commonly used conventional motor types and called outrunner BLDC with stators located at inner and rotor at the outer region of the motor. As seen in Figure 1(a), hub motors have a wheel form because they are designed for electrical or solar vehicles. The outer region of the rotor was designed as a tyre covered rim. Permanent magnets are aligned on the inner surface of the rotor. The stator part of the motor generally includes 3 coils as the 3-phase hub motor which was used in this study also had. Hub motors have hall effect sensors located near to the coils on the outer surface of the stator where they are triggered by permanent magnets. The driver unit triggers the coils, respectively, with the help of position based pulses of hall effect sensors. By the way, triggering time is varied with the rotating speed of the hub motor.  Figure 1(b) shows the electrical equivalent circuit of the hub motor, which helps to achieve the mathematical model of the hub motor.

The mathematical model of a hub motor is similar to that of a conventional BLDC as its electrical equivalent circuit is also similar. But some major physical differences change the performance and characteristics.

The mathematical model is expressed by

(1) vrn=Rir+pΨr+er,vsn=Ris+pΨs+es,vtn=Rit+pΨt+et

with

(2) vrn=vr−vn,vsn=vs−vn,vtn=vt−vn,

where *R* is stator resistance per coils *i*_*r*_, *i*_*s*_, *i*_*t*_ and Ψ_*r*_, Ψ_*s*_, Ψ_*t*_ are, respectively, phase currents of coils *r*, *s*, *t* and total flux linkage of *r*, *s*, *t* coils, *p* is the Laplace operator, and *v*_*rn*_, *v*_*sn*_, *v*_*tn*_ are, respectively, the voltages of coils *r*, *s*, *t* and neutral node. Flux expressions are given by

(3) Ψr=Lir−Mis+it,Ψs=Lis−Mir+it,Ψt=Lit−Mir+is,

where *L* is the self-inductance and *M* is the mutual inductance:

(4) $$\begin{array}{c} i_{r}+i_{s}+i_{t}=0. \end{array}$$

Substituting (4) in (3), we obtain

(5) Ψr=irL+M,Ψs=isL+M,Ψt=itL+M,Leq=L+M.

The model of the system in matrix form is

(6) $$\begin{array}{c} V=RI+pL_{eq}I+E, \end{array}$$

where

(7) I3=100010001,I=I3irisit,V=vrnvsnvtn,Ε=ereset.

Decoupled phase equations are obtained and explicit current equation systems are

(8) $$\begin{array}{c} p\left(\;\frac{I}{I_{3}}\;\right)=\left(\;\frac{1}{L_{eq}}\;\right)I_{3}\left[\;V-RI_{3}-E\;\right]. \end{array}$$

As it can be expressed from (1)–(8), most of the faults are directly related to resistance (*R*) and inductance (*L*). Voltage and current variables as measured parameters are affected by *R* and *L* values.

**Figure 2:**
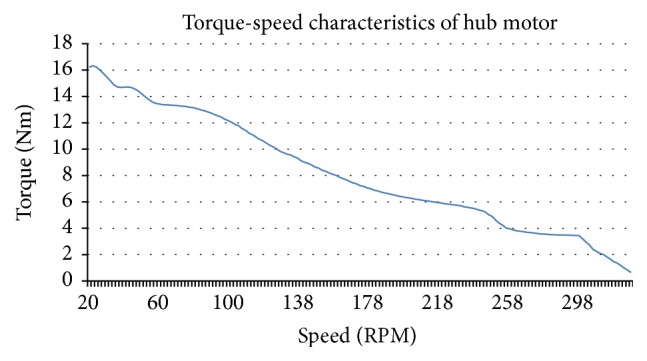
Torque-speed characteristics of hub motor.

Other measured parameters of hub motors for fault diagnosis are torque and speed variables. Mathematical expressions of torque and speed values are shown in (9) and (10):

(9) $$\begin{array}{c} T_{e}=\frac{e_{r}i_{r}+e_{s}i_{s}+e_{t}i_{t}}{n}, \end{array}$$

where *n* is the mechanical speed of the rotor and *T*_*e*_ is electromagnetic torque of hub motor:

(10) $$\begin{array}{c} \frac{dn}{dt}=\frac{T_{e}-T_{m}-Bn}{J}, \end{array}$$

where *T*_*m*_ is the mechanical torque, *B* is the damping constant, and *J* is the moment of inertia. As seen in (9) and (10), the speed parameter is related to the torque value and the torque parameter is related to the coil currents of the hub motor. Because of dynamic impact between faults and current, voltage, torque, and speed values, these variables were considered as parameters to be measured for a more successful fault diagnosis of the hub motor. Torque-speed characteristics of a hub motor are given in Figure 2.

As can be seen in Figure 2, the torque characteristics of the hub motor are appropriate for vehicles, this is because the take-off torque of the hub motor is high and vehicles need higher torque levels for acceleration at the starting time. Another reason for hub motors to be chosen for vehicles is the higher power efficiency according to other choices. Hub motors do not need any transmission or differential devices which cause mechanical losses. Hub motors also save more space by their compact structure because the motor is located inside the wheel.

## 3. Test Set and Data Acquisition

**Figure 3:**
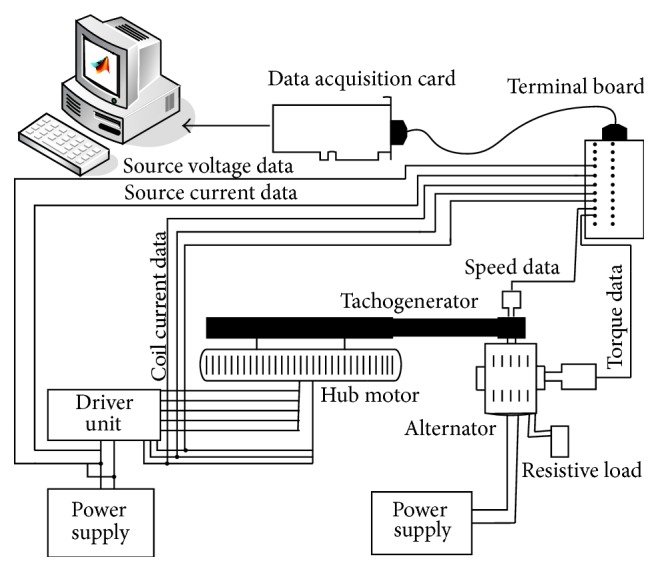
Hub motor data acquisition and test set block diagram.

Methodologies of condition monitoring, fault detection, and fault diagnosis systems are generally based on comparing measured abnormal variables with measured normal ones. Reliable and varied measurement of variables is important for more efficient fault diagnosis and condition classification. Therefore, experimental set design is also very important for a better monitoring, detection, and diagnosis system. An experimental test set was designed for this real-time process for fault diagnosis and monitoring of the hub motor. Block diagram of the data acquisition and test set for hub motor is seen in Figure 3.

**Figure 4:**
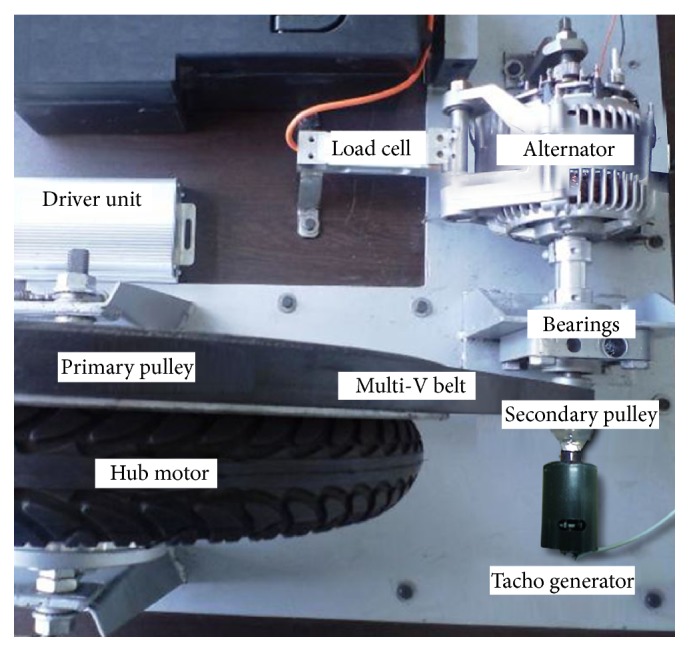
Hub motor data acquisition and test set.

The experimental set was designed in order to allow all 7 main parameters which were decided as input variables of the fault diagnosis system to be measured as seen in Figure 3. These variables are source voltage, source current, *r* coil current, *s* coil current, *t* coil current, the mechanical speed of the motor, and output torque of the motor.  Figure 4 shows a picture of the test set data acquisition system.

In this experimental set, the hub motor was loaded with a synchronous alternator connected to the hub motor via a belt and pulleys system as seen in Figure 4. The alternator was loaded with a resistive load and was excited with an adjustable power source which made the alternator behave as a retarding system. Multi-V type belt and pulleys were applied to the system that grips together better and prevents dislocation of the pulleys of the hub motor and alternator. The pulley system had two functions: one of these was to fix the rotating speed of the alternator to a significant level according to its characteristic rotating speed for a linear retarding power and the second one was to overcome the mismatch of the rotor and stator positions of the hub motor and alternator. The primary pulley was fixed at the same origin with the axle of the hub motor and rotated at the same speed with the hub motor. A secondary pulley was fixed at the same origin with the axle of the alternator. The diameter of the primary pulley was 16.5 times bigger than the secondary pulley, so the alternator rotates 16.5 times faster than the hub motor with a maximum speed of 5775 RPM. All data was acquired with the same frequency of 40 Hz.

**Figure 5:**
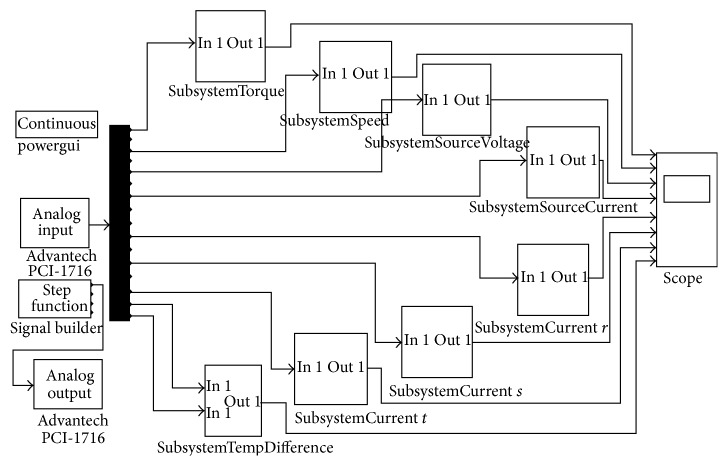
MATLAB Simulink model which was designed for data acquisition.

Figure 5 shows the designed MATLAB Simulink model for generating the tracing signal to drive the hub motor, acquiring and processing the data with some filters and embedded functions for significant and clear data, and multiplying them with calculated constants inside the subsystem blocks. The input voltage amplitude of coils traced the signal applied by signal builder of Advantech PCI-1716. The data input was supplied by analogue input of Advantech PCI-1716. It was possible to monitor data curves by the scope. The data was also stored for composing data set and using them to construct, train, and test the neural network by identical conditions and constants.

The hub motor's rotating speed data was acquired with a tachogenerator which generates a linear voltage amplitude level directly proportional to its rotating speed within the operation range. The tachogenerator was fixed to the axle of the secondary pulley for better precision because of the higher speed level. Speed data was acquired as voltage level and the speed value was converted to integers with an embedded function inside the “SubsystemSpeed” block as seen in Figure 5. Voltage data was filtered with a low pass filter to eliminate the noises and distortions and then multiplied with a calculated gain to convert the voltage to speed data as RPM. Source voltage as input voltage between power source terminals was applied to the data acquisition card to be measured with a degrading circuit for matching the input voltage limits of the data acquisition card. After this, the source voltage data was applied to a low pass filter and multiplied with the gain as the mathematical inverse of the degrading circuit constant inside “SubsystemSourceVoltage” block seen in Figure 5 to reachieve the original voltage data.

Source current as input current, which flows from the power source, *r* coil current, *s* coil current, and *t* coil current data were measured with current probes and were applied to a low pass filter and then multiplied with gain blocks regarding the mathematical inverse of conversion ratios of current probes.

For acquiring torque data, a synchronous alternator supplied retarding power by applying voltage to excitation input and was loaded with a resistive load. The alternator was fixed from front and rear parts of its axle and body of the alternator was located as to be rotating freely around itself in the air. A load cell was located on the body of alternator. The alternator loads the load cell when it was loaded with retarding power because of the voltage applied between excitation inputs. The distance between the axle of the alternator and the load cell was taken into account and was added into the “SubsystemTorque” block as a gain as seen in Figure 5. Torque data was also applied to an embedded function for eliminating possible negative torque values that can never be achieved but may suddenly be seen because of oscillations occurring while starting and stopping or in some unstable torque situations of the hub motor. The output of the load cell was connected to a load cell amplifier that amplifies the load cell output signals for achieving meaningful amplitudes of voltage signals for converting torque data as Nm. The load cell amplifier output signal was calibrated and applied to a data acquisition card. Inside the “SubsystemTorque” block in the MATLAB Simulink model, the signal was applied to a low pass filter and multiplied with the calculated gain. Also, the load cell and load cell amplifier were used as a torque sensor and torque data was acquired reliably.

The hub motor was driven with the designed tracing reference signal generated via the data acquisition card. The hub motor tracing signals were applied as sinusoidal, ramp, and step functions at various loading levels to create more and significant differences between the data signals of various conditions for faults to be diagnosed. After the measurements of all tracing signals, step function was determined for a better diagnostic. Differences between the determined situations were more significant under the step function tracing signal. When the sinusoidal signal or ramp signal was used as a reference signal to be traced, data classification was more difficult because of varied signal amplitudes which prevent grouping of the situation according to signal amplitude. It might be possible to process the procedure by taking the average of all of the data and then begin the fault diagnosis algorithm. But it would prevent real-time operation or it may cause a lagging for the output and it would decrease the accuracy because of a lower number of diagnosis processes for the time interval to be diagnosed.

**Figure 6:**
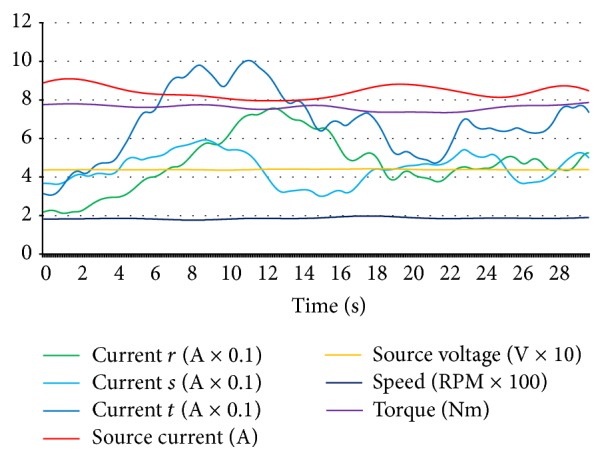
Normal condition data curves of hub motor.

The half-loaded situation was used for a better solution in the neural network training after some experimentation for composing data set. Because, when the hub motor was not loaded, symptoms of some faults were waned and when the motor was loaded at the maximum level, the hub motor could not continue rotating for some fault situations and stopped allowing data acquisition for diagnostics. That is why the data set was prepared for step function tracing with a maximum level of driving amplitude and half loaded motor. In Figure 6, data curves of hub motor for normal conditions are shown.

**Figure 7:**
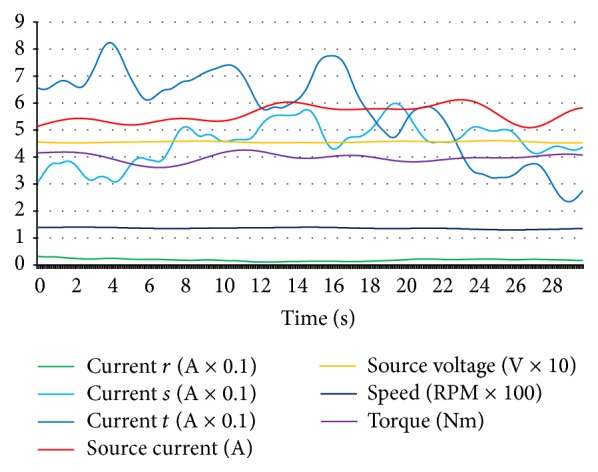
*r* coil open circuit fault data curves of hub motor.

As seen in Figure 6, values are suitable for designing a neural network because the data is clear and suitable for being classified. The normal condition data of the hub motor was stored while the motor was in a healthy situation and, in Figure 7, curves for the open circuit fault of coil *r* are given with identical classification and configuration with Figure 6.

**Figure 8:**
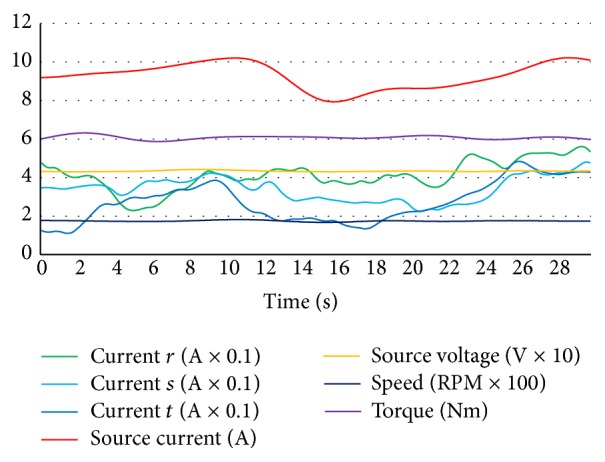
*r* coil short circuit fault data curves of hub motor.

For generating an open circuit fault, *r* coil was forced to be nonconductive by making an open circuit in *r* coil and data was stored for composing data set for *r* coil open circuit fault. As it can be seen clearly in Figure 7, the current values of *r* coil decreased to zero level and source current values also decreased. On the other hand, the speed value decreased and torque was unstable with respect to normal condition values. *s* and *t* coils open circuit faults had identical characteristics for source current, source voltage, torque, and speed level, but when *s* coil was open circuit, *s* coil current was at zero level with increased *r* and *t* coil currents and, in *t* coil open circuit, *t* coil current was at zero level with increased *r* and *s* coil currents as expected.  Figure 8 shows the short circuit fault data of *r* coil.

**Figure 9:**
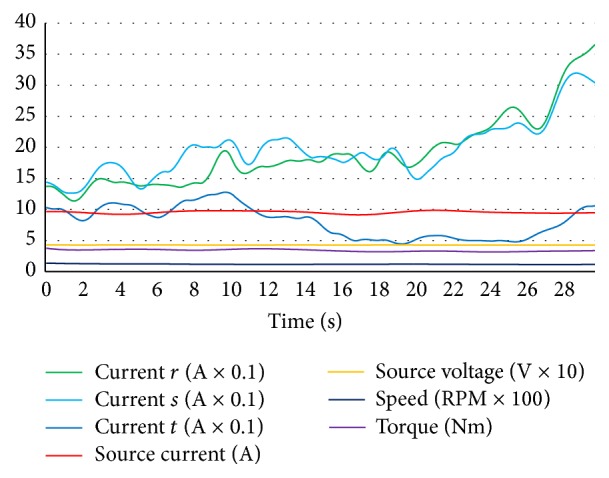
Short circuit between *r* coil and *s* coil fault data curves of hub motor.

An adjustable 1 kW carbon resistor which was adjusted to 2 Ω was connected in parallel with *r* coil and was forced to generate short circuit characteristics for acquiring and storing *r* coil short circuit data as seen in Figure 8. Short circuit resistance less than 2 Ω caused higher current flow through the driver circuit and that caused the fuse to cut off for instantaneous current protection. More hazardous current levels can also be detected without the need of any fault diagnosis system by identifying the fuse cutting off. And this fault diagnosis system must also diagnose faults from the beginning level for preventing more hazardous faults as a preventive protection. If the fuse was deactivated for identifying higher currents of a short circuit (although the generated short circuit was hazardous and high enough), it was confidently more easier and possible for this fault diagnosis system to diagnose short circuit current which was higher than this level and it should not be forgotten that hub motors are also being operated with fuses in real operation to protect them and the system is feasible with diagnosing faults from the beginning level in real operation for fault diagnosis and preventive fault protection. So fuses must not be deactivated because of the fault diagnosis process. Short circuit fault of *r* coil caused higher source current and lower torque level. And *r* coil current was higher than *s* and *t* coil currents. Short circuit for *s* and *t* coil faults had the same values for source current and voltage, torque, and speed variables. But one coil short circuit faults could be distinguished and classified by identifying the differences in coil current values. In Figure 9 short circuit between *r* and *s* coils fault curves are shown.

**Figure 10:**
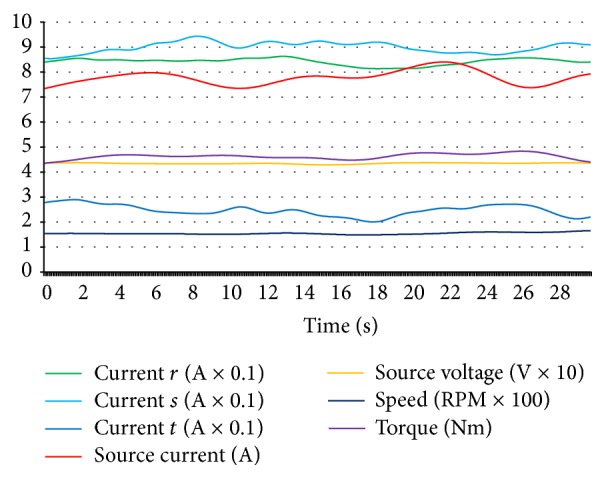
*r* coil sensor fault data curves of hub motor.

For a short circuit fault to be realized between *r* and *s* coils, a short circuit wire with a 4 Ω resistor was connected between *r* and *s* coils for characterizing short circuit fault between the coils. A lower resistive value of short circuit resistors would cause fuse cutting off because of the higher current overflow. When the short circuit between *r* and *s* coils fault occurred, the *r* and *s* coil currents increased to a higher level and the source current was also very high. Torque level decreased and the speed of the hub motor was very low as it can be analysed in Figure 9. Short circuit between *r* and *t* fault and short circuit between *s* and *t* fault had identical characteristics for source current, source voltage, torque, and speed variables, but distinguishing the difference between short circuit faults for 2 coils occurred at coil current values. By Figure 10, *r* coil sensor fault data curves can be analysed.

**Figure 11:**
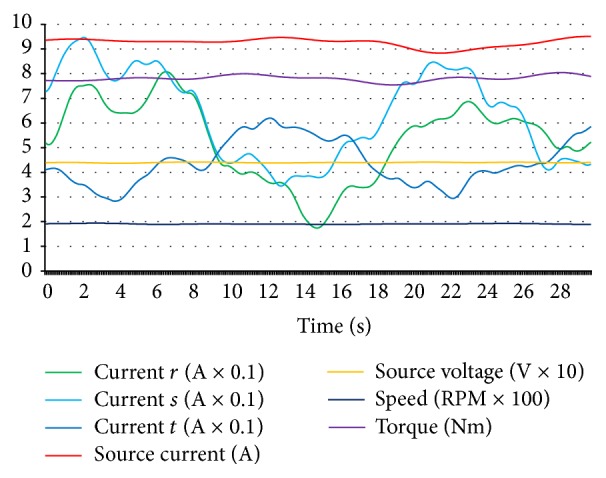
Bearing fault data of hub motor.

For generating an *r* coil sensor fault, *r* coil sensor output cable was disconnected as *r* coil sensor could fail. Data was acquired from this situation to save, monitor, and compose *r* coil sensor fault part of data set. Figure 10 shows that currents of *r* and *s* coils were greater and that of *t* coil was lower with respect to *r* and *s* coil current values. The lower level of torque and speed values were the featured characteristic properties of *r* coil sensor faults of the hub motor. *s* coil sensor fault and *t* coil sensor fault had also the same characteristics for speed, torque, source voltage, and source current values. Varied *r* coil current, *s* coil current, and *t* coil current made the difference to be distinguished for which sensor faults occurring among sensor faults. Bearing fault data curves of the hub motor are shown in Figure 11.

For generating a bearing fault, one of the healthy shaft bearings of the hub motor was demounted, then a used and failed bearing was replaced instead of a healthy one, and then data was acquired for a bearing fault of the hub motor as seen in Figure 11. Source current and all coil current values increased and torque value decreased at the bearing fault.

As explained for Figures 6–11, all possible 14 conditions for hub motors had specific characteristics and they can be distinguished by enough acquired data. Related faults of all coils can be distinguished by the coil currents, and “open circuit faults” group, “short circuit faults” group, “short circuit between coils faults” group, “sensor faults” group, “bearing faults,” and “normal condition” situations can be distinguished by varied speed, source voltage, source current, and torque data. If this study had not aimed at a precise and detailed fault diagnosis, the coil short circuit faults for all coils would have been grouped under a general name of “open circuit fault”; currents of each coils would not have been needed to be acquired. But this study aims at a complete and detailed diagnosis of all possible faults of the hub motor. That is why, all 7 input data which were acquired were vital for fault diagnosis for this system.

## 4. Constructing and Training the Artificial Neural Network

With the emerging technology of artificial neural networks, human expertise can be mimicked and automated to a certain extent. Feedforward neural networks can be trained to perform motor fault detection by learning the experts' knowledge using a representative set of motor data [38], as well as the fact that a feedforward neural network was chosen for this real-time monitoring and fault diagnosis system for higher compatibility and success levels.

Kenneth Levenberg and Donald Marquardt developed the LM (Levenberg-Marquardt) Algorithm [39, 40], which provides a numerical solution to the problem of minimizing a nonlinear function [41]. When backpropagation algorithms were compared between each other, LM algorithm has been found to be faster and to have better performance than the other algorithms (CGF (Conjugate Gradient with Fletcher-Reeves) and RB (Resilient Backpropagation)) in training [42]. Considering these advantages, LM training algorithm was chosen to be used in this study.

A feedforward neural network has input layer, output layer, and one or more hidden layers. The neural network was chosen to be constructed with one hidden layer. However, if too many hidden neurons are used, overfitting can occur, that is, although the network can be trained to work very well for the training data, it performs poorly for test data not used in training, or, in other words, the network is not able to generalize well [43]. It is essential to optimize the success percentage of the neural network with effectual numbers of neurons for the hidden layer to achieve the best performance level.

**Figure 12:**
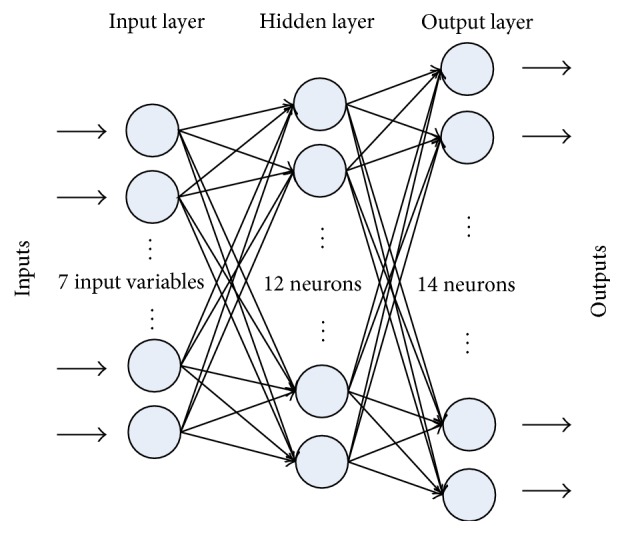
General structure of constructed feedforward backpropagation neural network.

Various numbers of neurons were tried based on experiences about neural networks within the estimated limits and successes of the neural networks were compared to achieve the best success percentage for the neural network. The most suitable neural network neuron numbers combination was determined as seen in Figure 12 as the summarized and general structure of optimized feedforward neural network which was designed and constructed.

**Figure 13:**
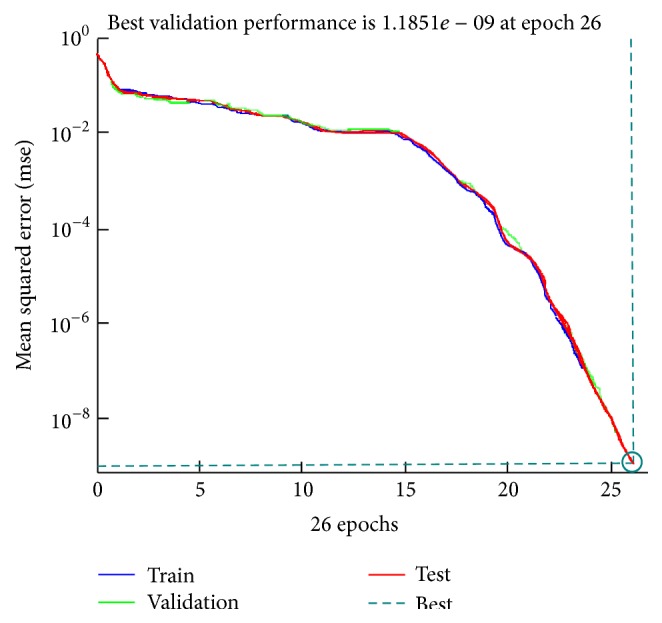
Training performance curves of neural network by MATLAB.

The artificial neural network was constructed with 7 input variables for input, 12 neurons for hidden layer, and 14 neurons for output layer with the same number of all possible conditions of the hub motor as chosen to be the number of outputs for this real-time fault diagnosis system as seen in Figure 12.  Figure 13 shows the training performance curves of neural network training process by MATLAB.

**Figure 14:**
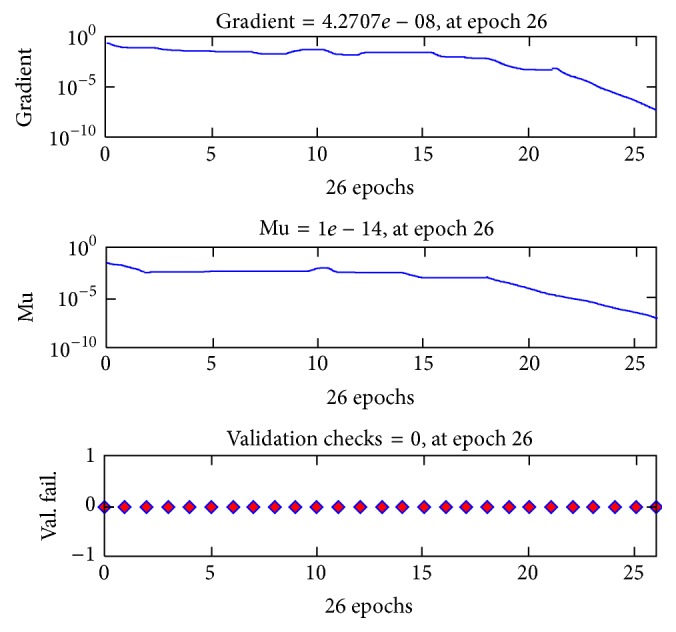
Training state data of neural network by MATLAB.

Figure 13 indicates the best validation performance of the neural network and shows training, validation, and test curves of the training process. Minimum gradient was decided as 1 × 10^−7^ for the goal in the training process. The training has reached the goal at the 26th epoch. The training state throughputs during 26 epochs of neural network are shown in Figure 14.

Figure 14 indicates how the gradient and mu (weight changes of neural network) change and number of validation checks during the 26 epochs of neural network training process. These outputs of training process indicate a successful state of neural network.

## 5. Real-Time Fault Diagnosis Using Feedforward Backpropagation Neural Network

**Figure 15:**
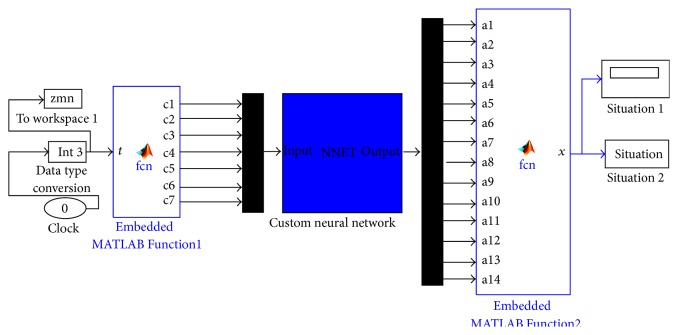
MATLAB Simulink model for neural network performance test.

Neural network was trained with previously acquired 4160 sets of data. After the neural network was constructed and trained, it was necessary to examine the success level of the neural network. For this purpose, a MATLAB Simulink model was designed as seen in Figure 15 for the neural network performance tests.

**Figure 16:**
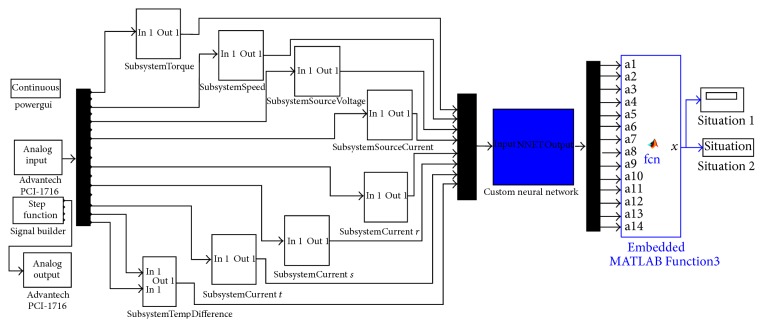
Neural network integrated real-time fault diagnosis system by MATLAB Simulink model.

The data set which was applied to train feedforward backpropagation neural network and outputs were delivered with the embedded MATLAB function as shown in Figure 15. Coded and embedded MATLAB function on the left side of Figure 15 applied the input sets one by one to the neural network to decide the condition and to diagnose faults. Neural network outputs were applied to another embedded function on the right side of Figure 15 which deals with the output according to the motor situation. All inputs and outputs were saved to the workspace on MATLAB. And via another m-file coded in MATLAB, outputs of the neural network were compared with true diagnostics. This m-file revealed the success performance of the neural network. Success percentage of the neural network with the training data set was revealed as 100%. Then, the neural network was tested with the same algorithm as seen in Figure 15 by another real-time acquired data set. It was decided that data set for testing must be at least 25% in width of the training data set. That is why 1040 sets of data which were not used in the training process were used for this success percentage test. The neural network again revealed 100% successful output as a result.  Figure 16 shows the neural network integrated real-time fault diagnosis system.

Then the data was acquired and processed in real-time with a real operating hub motor, in the same way as was done when the data set was being composed. While the hub motor was being operated, all 14 conditions, composed of 13 faults and 1 normal (healthy) situation, were forced to be realized. The hub motor was tested under these 14 different conditions homogenously 100 times each which had hundreds of snap data and diagnosis with 40 Hz of acquiring and diagnosing frequency in real-time and neural network integrated real-time fault diagnosis system which is seen in Figure 16 revealed more than 99% success. Tests were realized with 99 continuous stable true diagnostics. When the bearing fault was being diagnosed, the fault diagnosis system revealed an unstable output changing between highly rated true diagnostics with “Bearing Fault” output and rarely with “Normal Condition” (healthy) output. This result was counted as a false diagnostic because of the unstable output during 10 seconds of testing time with 40 Hz diagnosis frequency. If a more damaged bearing was mounted on the motor while bearing fault diagnostic was being tested, the system would also diagnose this situation successfully and the test would have been achieved with a 100% success rate. This diagnostic about the bearing fault depends on the damaging level of the bearing used for tests.  Table 1 shows the test results and success percentage of the fault diagnosis system.

**Figure 17:**
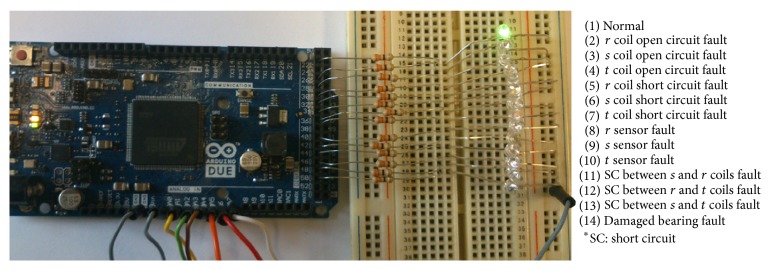
Mobile prototype of the real-time monitoring and fault diagnosis system.

After the high rated success results which can be seen in Table 1, a decision was reached to produce an applicable and mobile prototype of the fault diagnosis system. For this reason, a microcontroller embedded Arduino Due card was used for prototype application as seen in Figure 17 because of its economic, compact, and applicable structure for mobile and PC-free fault diagnosis system.

The fault diagnosis system which is seen in Figure 16 and designed for an Advantech data acquisition card was modified for Arduino Due and the input ranges inside the subsystems were also adapted for Arduino Due. Then the adapted system was embedded into a microcontroller circuit. In Figure 17, microcontroller embedded circuit was connected to the hub motor in normal condition. It was energized and 7 input parameter and ground cables were connected below the Arduino Due microcontroller circuit card and on the right side of the circuit, ground cable and 14 microcontroller embedded prototype circuit outputs of the real-time monitoring and fault diagnosis system for all situations were connected to LEDs. So the operator can see the situation or fault clearly by the on/off positions of the LEDs. By the way, a mobile and compact prototype of the real-time monitoring and fault diagnosis system which can be used in vehicles with a hub motor was designed and manufactured.

## 6. Results and Conclusions

All possible faults were diagnosed for hub motors by a designed feedforward backpropagation neural network embedded fault diagnosis system. These are *r* coil open circuit, *s* coil open circuit, *t* coil open circuit, *r* coil short circuit, *s* coil short circuit, *t* coil short circuit, *r* hall effect sensor, *s* hall effect sensor, *t* hall effect sensor, short circuit between *s* and *r* coils, short circuit between *s* and *t* coils, short circuit between *r* and *t* coils, and damaged bearing faults. Besides these, the normal condition of the hub motor was also detected by the system. While determining the faults of the hub motor, all probabilities that were realized as faults of a hub motor were considered and all these 14 situations were determined by the help of fault diagnosis literature and previous experiences. Most of various studies about fault diagnosis were generally concerned about only one or some parts of all faults. But this study defeats a gap in fault diagnosis literature of hub motors by diagnosing all possible faults of a hub motor with a comprehensively generated neural network. This study will also increase the number of studies about hub motors, because not enough studies have been carried out about monitoring and fault detection or the diagnosis of hub motors.

This fault diagnosis process might be realized with a lower number of input variables with a lower rate of success percentage, with 2 or more input variables just as torque and speed data. This would let us diagnose a lower number of faults with a low level of success percentage ratio. But this study supplies all possible faults of the hub motor to be diagnosed in real-time with a success percentage of 99% in real-time operation. A success percentage ratio of 99% in real-time for all 14 different outputs was achieved by dint of acquiring all essential 7 different input variables.

By this fault diagnosis system, hub motors will have more widespread usage because it will be more confident to use a hub motor because of preventing or early detection of some possible faults and also diagnosis of all faults. Diagnosis of faults in real-time will save the motor from getting more damage by preventing unaware driving with faults. Therewithal, quick diagnosis of faults will decrease the percentage of downtime suffered by hub motors with quick shift to mending or problem solving stages. Some faults that cannot be noticed or considered manually can also be diagnosed by the fault diagnosis system and these diagnostics will increase power efficiency of the hub motor in general, by preventing faulty usage of the hub motor. Because faulty conditions generally allow the motor to operate for a while until the fault gets big enough by means of fault effects to prevent operating of the motor, thereby, the mending stage will be faster and less costly with an early detected and diagnosed lower fault level. The system is able to diagnose all possible faults from the beginning levels of faults, while the motor is still operating.

This real-time fault diagnosis and condition monitoring system has a success percentage of 99% for real-time application and test. This success percentage was achieved at the most efficient point of the system, at a half loaded condition, and may decrease slightly for varied speed or load conditions. The success percentage can also be fixed stable at 99% by adding another load cell and an inclinometer to the system with an I/0 switch to apply diagnosis under suitable conditions, or it is also possible to switch the neural networks designed for light loaded, half loaded, and heavy loaded conditions via the same additive sensors for mobile applications. It is possible to improve the system and develop for various load conditions or various types and power ranges of hub motors. This initiator, feasible, and applicable system is not affected by transient conditions because of its fast responding design. This system was designed as ready to use and can successfully be applied with the current form.

A mobile prototype of the real-time monitoring and fault diagnosis system which was manufactured with a microcontroller embedded circuit may also easily be improved with a display or screen instead of LEDs for output, for serial manufacturing of this system. By the prototype, the system saves more space and energy for vehicles. By the guidance of this study, trip computer system that diagnoses the faults gives data output about the remaining distance which is possible to cruise with the remaining charge level of battery and gives the performance and condition data output about cruising can be developed for electrical vehicles which use hub motors. It can also be possible to switch the action to stop the motor after diagnostics of hazardous and urgent faults or only monitoring the diagnostics of faults as a warning. This study helps to increase the usage of renewable and electrical energy for vehicles as supporting hub motors by strengthening them with a confidential and successful real-time fault diagnosis system. This real-time, reliable, and compact fault diagnosis system presents an initiator, economical, and successful solution for electrical vehicles with hub motors.

## Figures and Tables

**Table 1:** Test results for fault diagnosis.

Test type	True diagnostics	False diagnostics	Success percentage
Data used in training	4160	0	100%
Data not used in training	1040	0	100%
Real time	99	1	99%
